# Supplementary material for: SARS-CoV-2 genomic and subgenomic RNAs in diagnostic samples are not an indicator of active replication
Source: Nat Commun. 2020 Nov 27;11:6059. doi: 10.1038/s41467-020-19883-7 (PMC7695715; doi:10.1038/s41467-020-19883-7)

Supplementary Information

SARS-CoV-2 genomic and subgenomic RNAs in diagnostic samples are not an indicator of active replication

Soren Alexandersen[1,2,3,*], Anthony Chamings [1,2], and Tarka Raj Bhatta [1,2,]

[1]Geelong Centre for Emerging Infectious Diseases, Geelong, VIC 3220, Australia; [2]Deakin University, Geelong, VIC 3220, Australia; [3] Barwon Health, University Hospital Geelong, Geelong, VIC 3220 Australia

* Correspondence: soren.alexandersen@deakin.edu.au; Tel.: +61-0-342159635

Supplementary Table 1. Table showing the position of the forward primer in each of the two Ampliseq pools as well as the predicted amplicon length from virus genome RNA and the individual subgenomic RNAs. Short subgenomic amplicons are indicated in bold type.

Forward primer	Forward primer 3'-end	Virus genome RNA	S Orf2	Orf3a	E Orf4	M Orf5	Orf6	Orf7a	Orf7b	Orf8	N Orf9	Orf10
Pool 1	41	188	265	**120**	143	170	**83**	237	239	224	**105**	226
Pool 2	51	225	**127**	198	**88**	**85**	161	**85**	**75**	**49**	221	**17**

Supplementary Table 2. Table showing the number of reads at MAPQ 32 with partial leader sequence in each sample mapped to the 5'-UTR, known subgenomic RNAs in SARS-CoV-2 and unknown/unrecognised TRS sequences.

	Read Mapping Location											
**Sample**	**Unknown TRS**	**5' UTR**	**S Gene**	**ORF 3a**	**E Gene**	**M Gene**	**ORF6**	**ORF 7a**	**ORF 7b**	**ORF 8**	**N Gene**	**ORF10**
**GC-28/67**	0	0	0	0	0	0	0	0	0	0	0	0
**GC-26/66**	4852	136473	1915	12813	13078	5852	4397	18731	73	1238	14155	0
**GC-13/35**	25	75	1	12	13	10	11	55	0	58	56	0
**GC-11/34**	439	854	5	21	52	44	36	508	0	850	23	0
**GC-11/38**	409	11889	158	1707	962	1589	988	3843	7	527	3215	0
**GC-20/63**	0	57	0	0	75	2	564	0	0	0	0	0
**GC-24/61**	1	575	0	1	10205	292	1	4766	0	0	9694	0
**GC-12/36**	1	0	0	3	0	0	1	1	0	1	1	0
**GC-14/33**	68	455	15	44	35	89	40	222	0	149	148	0
**GC-14/37**	2138	14857	362	4431	3374	5085	4047	11493	37	2155	20117	0
**GC-23/60**	408	7155	268	73	31556	424	7576	8801	0	2764	4282	0
**GC-51/62**	1	310	0	0	0	90	0	19112	0	0	11353	0
**GC-21/64**	10859	3680	0	0	0	85	0	8725	0	2431	3092	0
**GC-25/65**	25675	29936	11	9	10882	1174	9756	150151	0	27303	1226	0
**GC-55/68**	6345	8934	15	13	20126	3975	2777	152619	0	9242	587	0

Supplementary Table 3. Table showing the number of reads containing the partial leader sequence when searched for in the following SARS-CoV-2 Short Read Archives (SRA)'s in NCBI's SRA database. The number of reads with partial leader sequence mapped from each SRA mapped to the 5' UTR, subgenomic RNAs and unknown/unrecognised TRS sequences are shown.

SRA	Sequencing Platform	Sample	Library Creation	Unknown TRS	UTR	S Gene	ORF 3a	E Gene	M Gene	ORF 6	ORF 7a	ORF 7b	ORF 8	N Gene	ORF 10	Total Subgenomic reads	Total Reads with Leader
**ERR4157962**	Illumina	Culture	Amplicon	14	0	69	17	14	56	52	36	0	7	188	0	439	453
**ERR4157960**	Illumina	Culture	Amplicon	10	1451	82	10	4	47	89	10	0	0	285	0	527	1988
**SRR11267570**	Nanopore	Culture	RNA Seq	27	26	4	32	4	142	53	171	6	147	1395	0	1954	2007
**SRR11648002**	Nanopore	Clinical	Amplicon	2	2817	0	0	7	0	1	0	0	0	1	0	9	2828
**SRR11648052**	Nanopore	Clinical	Amplicon	15	2194	2	0	8	0	0	1	0	0	18	0	29	2238
**SRR11779980**	Illumina	Culture	RNA Seq	1	59	2	5	3	2	1	6	0	2	16	0	37	97
**SRR11779998**	Illumina	Culture	RNA Seq	1	901	9	29	5	4	14	21	0	8	50	0	140	1042
**SRR11810731**	Ion Torrent	Clinical	Amplicon	1	1935	0	78	328	719	0	1120	0	0	169	0	2414	4350
**SRR11810737**	Ion Torrent	Clinical	Amplicon	299	4692	313	1551	1075	1989	544	4138	37	402	4954	0	15003	19994
**SRR11528306**	Illumina	Culture	Amplicon	0	0	0	0	0	0	0	1	0	1	1	0	3	3
**SRR11454612**	Illumina	Clinical	RNA Seq	0	0	0	0	0	0	0	0	0	0	0	0	0	0
**SRR11454615**	Illumina	Clinical	RNA Seq	0	6	0	0	0	0	0	0	0	0	2	0	2	8
**SRR11454609**	Illumina	Clinical	RNA Seq	0	73	0	4	0	0	3	0	0	1	2	0	10	83
**SRR11178050**	Nanopore	Clinical	Random primer	0	0	0	0	0	0	0	1	0	0	0	0	1	1
**SRR10948474**	Nanopore	Clinical	RNA Seq	22	137	5	19	1	11	2	19	0	4	16	0	77	236

Supplementary Table 4. Table showing sample details with corresponding Ct value of strand specific PCR amplification using specific targets

Sample	Sample collection date	cDNA FP2<br><br>Negative strand<br><br>7a Genomic and Sub-genomic<br><br>(Set 2) (Ct)	cDNA RP1-3<br><br>Positive strand 7a Genomic and sub-genomic<br><br>(Set 2) (Ct)	cDNA P1<br><br>Negative strand Leader-7a Subgenomic only<br><br>(Set 1) (Ct)	cDNA RP1-3<br><br>Positive strand Leader-7a Subgenomic only<br><br>(Set 1) (Ct)	cDNA FP2<br><br>Negative strand 7a Genomic and sub-genomic (Set 5) (Ct)	cDNA RP1-2<br><br>Positive strand 7a Genomic and sub-genomic genomic<br><br>(Set 5) (Ct)
NTC		Neg	Neg	Neg	Neg	Neg	Neg
GC-26/66	7/03/2020	Neg	**23.4**	Neg	**28.6**		
GC-13/35	23/03/2020	Neg	**28.8**	Neg	Neg		
GC-11/34/38	24/03/2020	Neg	**19.7**	Neg	**24.8**	Neg	**20.1**
GC-14/33/37	28/03/2020	**29.6**	**21**	Neg	**26.7**	No Ct but correct Tm	**21.5**
GC-23/60	8/04/2020	Neg	Neg	Neg	**29.5**		
GC-25/65	10/04/2020	**27.6**	**19**	**28.5**	**23.5**		
GC-55/68	24/04/2020	**28.6**	**19.8**	**30.5**	**25.5**		

Note: NTC, non-template control (water). Positive results are indicated in bold and underlined for negative strand positivity while negative strand positivity for the 7a subgenomic RNA is further indicated in red.

Supplementary Table 5. Table showing volume adjusted PCR Ct values for fractions of samples GC-26/66 and GC-55/68, respectively, subjected to fractionation to look at membrane protection of SARS-CoV-2 RNAs (Non-Triton treated fractions only)

Fraction Treatment					PCR Results					
Sample Fraction	1000 x g	Triton	Nuclease	10,000 x g	ORF1ab	S	N	7a (Set 2)	Leader-7a (Set 1)	7a-plus sense
GC-26/66 P1P10T-N+	Pellet	No	Yes	Pellet	18.2	17.8	21.3	15.9	20.0	18.5
GC-26/66 P1S10T-N+	Pellet	No	Yes	Supernatant	22.1	21.9	25.2	20.8	24.4	23.0
GC-26/66 P1P10T-N-	Pellet	No	No	Pellet	19.5	19.4	22.8	18.0	22.1	21.0
GC-26/66 P1S10T-N-	Pellet	No	No	Supernatant	21.9	21.8	25.1	21.1	25.3	22.4
GC-26/66 S1P10T-N+	Supernatant	No	Yes	Pellet	24.8	24.7	28.5	23.9	29.4	Neg
GC-26/66 S1S10T-N+	Supernatant	No	Yes	Supernatant	27.9	27.2	31.3	26.6	Neg	Neg
GC-26/66 S1P10T-N-	Supernatant	No	No	Pellet	20.7	20.3	23.7	19.7	23.7	21.7
GC-26/66 S1S10T-N-	Supernatant	No	No	Supernatant	22.3	22.0	25.3	21.9	25.3	23.2
GC-55/68 P1P10T-N+	Pellet	No	Yes	Pellet	30.1	24.0	28.5	25.6	27.7	Neg
GC-55/68 P1S10T-N+	Pellet	No	Yes	Supernatant	31.1	32.0	29.9	25.3	30.0	Neg
GC-55/68 P1P10T-N-	Pellet	No	No	Pellet	17.8	17.3	21.9	15.8	20.3	18.9
GC-55/68 P1S10 T-N-	Pellet	No	No	Supernatant	20.1	19.8	23.9	18.4	21.9	20.8
GC-55/68 S1P10T-N+	Supernatant	No	Yes	Pellet	17.6	17.4	21.5	11.4	22.4	14.4
GC-55/68 S1S10T-N+	Supernatant	No	Yes	Supernatant	17.8	17.5	21.6	15.0	21.0	21.9
GC-55/68 S1P10T-N-	Supernatant	No	No	Pellet	16.3	16.5	19.9	13.0	18.8	16.6
GC-55/68 S1S10T-N-	Supernatant	No	No	Supernatant	16.2	16.3	20.2	12.8	17.6	16.5

Supplementary Figure 1. Violin plot showing the number of reads per total of 5 million reads at MAPQ 32 with the partial leader sequence which mapped to the start of a known subgenomic RNA in each sample. The median read count is indicated by the white dot, the interquartile range (IQR) by the thick bar, and the furthest values within 1.5*IQR indicated by the thin black line (n = 14 data points from 12 biological samples from 8 individuals run once).

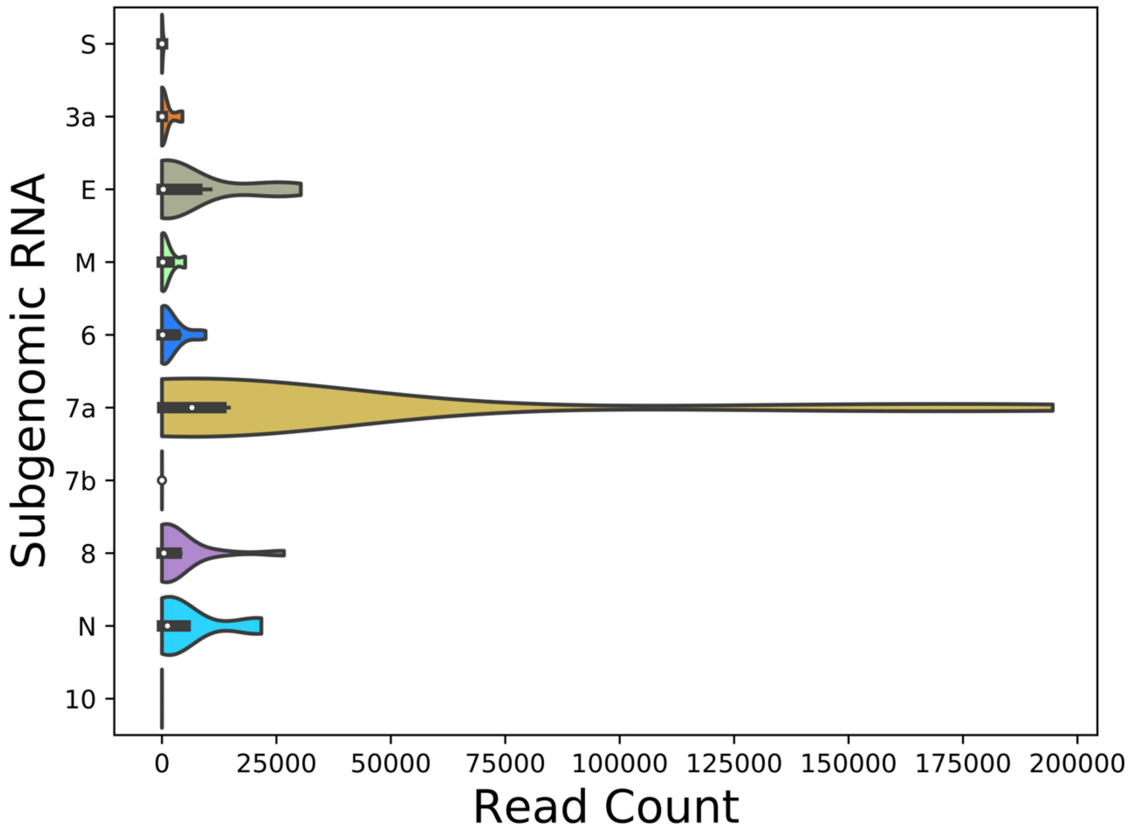

Supplementary Figure 2. Violin plot showing the estimated ratio of virus genomic reads to subgenomic reads containing the leader for each of the diagnostic samples included in the study detailing the ratios estimated by comparing (B) the most abundant amplicon in the first 21500 nt of the virus genome with the most abundant subgenomic RNA amplicon reads; (C) the most abundant full virus genome amplicon with the most abundant subgenomic RNA amplicon reads; and (D) the average full virus amplicons reads with the average subgenomic RNA amplicons reads. The median read count ratio is indicated by the white dot, the interquartile range (IQR) by the thick bar, and the furthest values within 1.5*IQR indicated by the thin black line (n = 14 data points from 12 biological samples from 8 individuals run once).

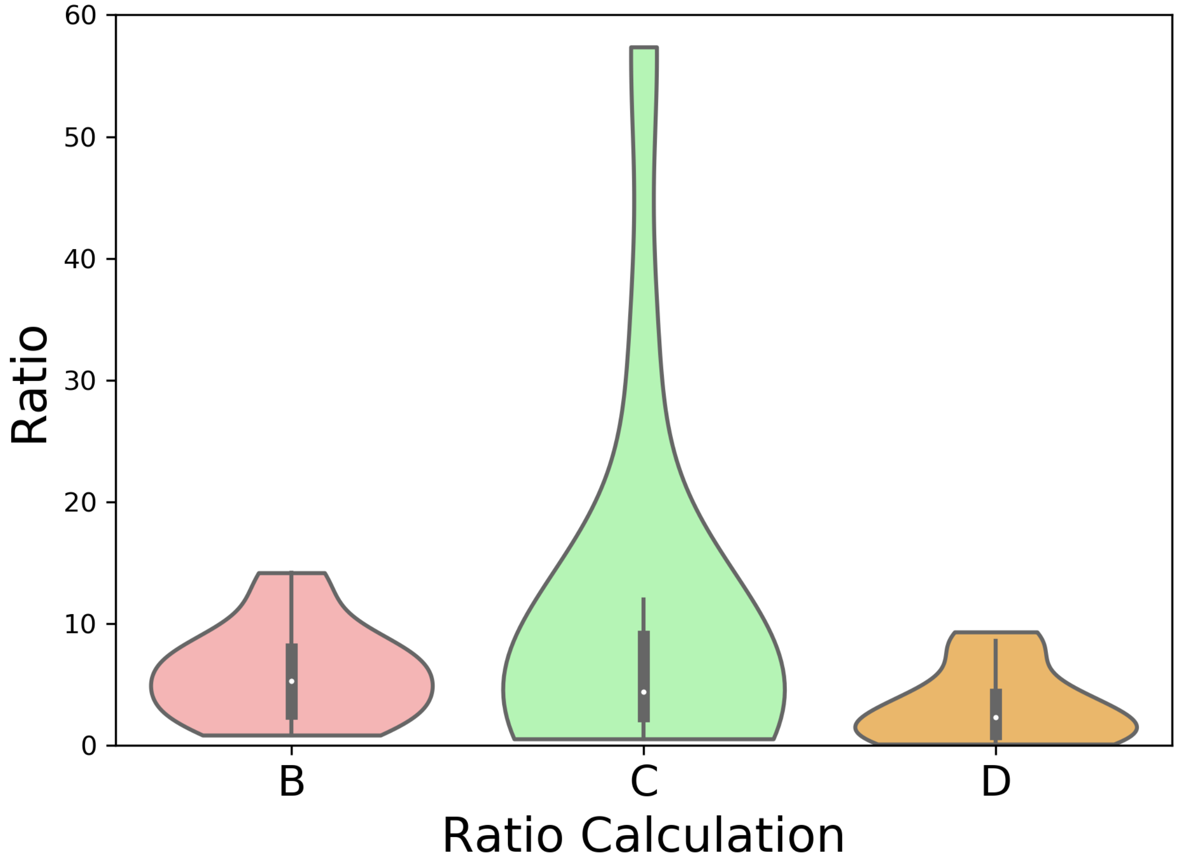

Supplementary Figure 3. Violin plot showing the estimated ratio of virus genomic reads to subgenomic reads containing the leader for each of the diagnostic samples included in the study detailing the ratios estimated by comparing (B) the most abundant amplicon in the first 21500 nt of the virus genome with the most abundant subgenomic RNA amplicon reads; and (D) the average full virus amplicons reads with the average subgenomic RNA amplicons reads. The median read count ratio is indicated by the white dot, the interquartile range (IQR) by the thick bar, and the furthest values within 1.5*IQR indicated by the thin black line (n = 14 data points from 12 biological samples from 8 individuals run once).

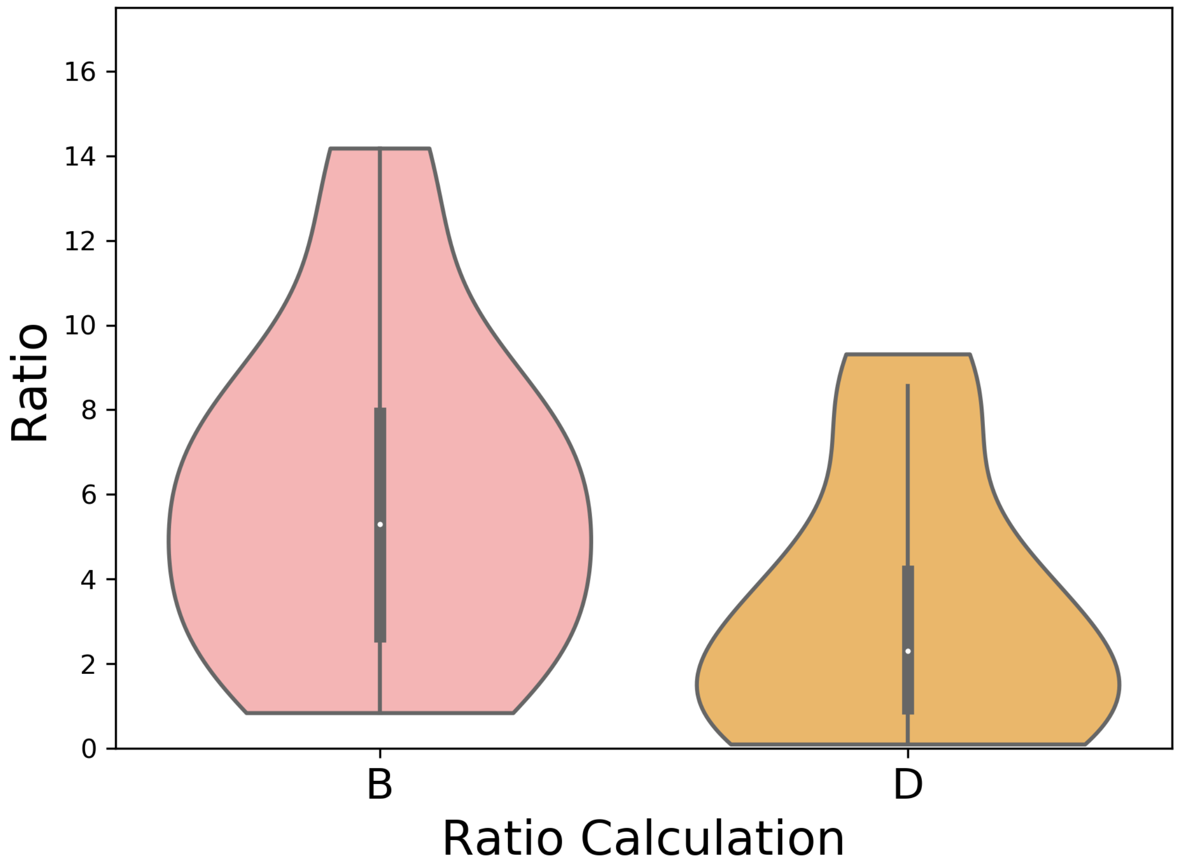

Supplementary Figure 4. Plots of PCR Ct values comparing the two PCRs detecting either the 5'-UTR genomic RNA only or the 7a total RNA (i.e. genomic RNA and subgenomic RNA up to and including 7a) respectively on the X-axes and the PCR Ct values for the 7a subgenomic RNA only on the Y-axes. The plots only include the values for the 7 samples having clear positive results in all three PCRs. As can be seen from this plot, the correlation is near linear and very close correlated (slope of 1.02 and 0.93 respectively and R^2 for both of 0.99). The differences in Ct values of the two PCR comparisons are close to 2.3 and 5.2 Ct, respectively, which at the efficiency of these PCRs equals around 4- to 20-fold more genomic or 7a total RNA compared to 7a subgenomic RNA only.

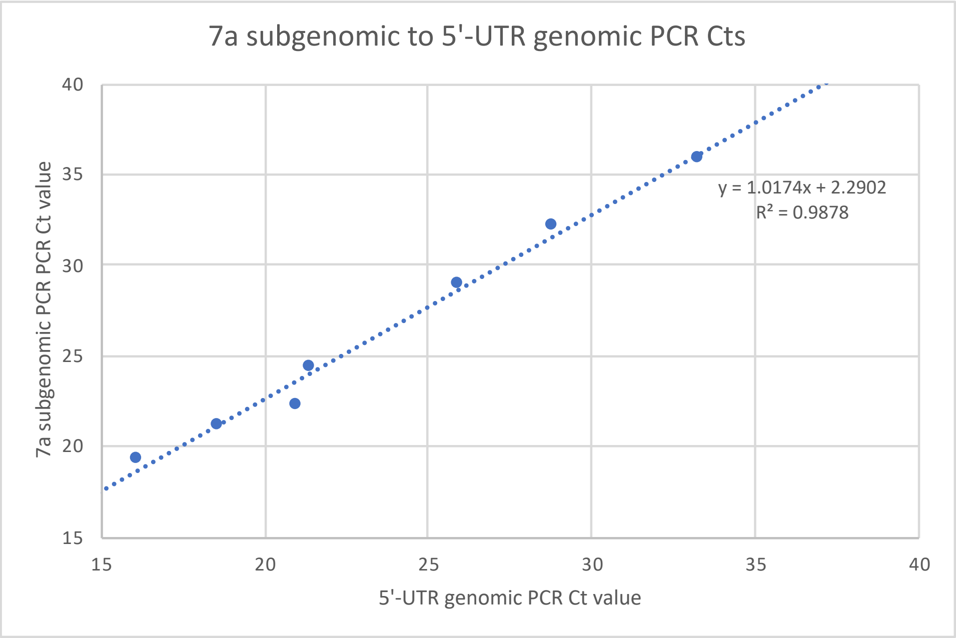

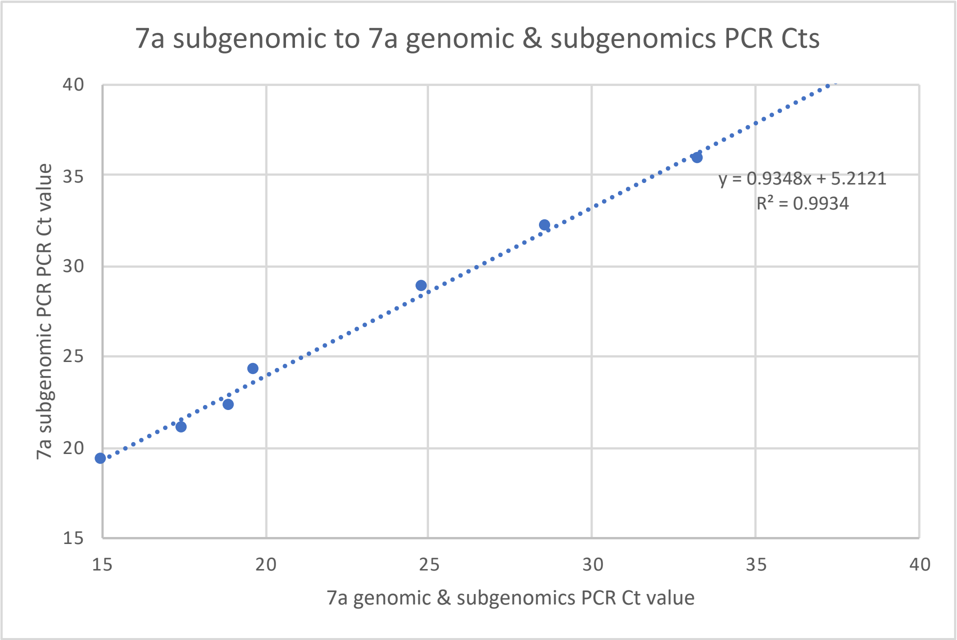

Supplementary Figure 5. Volume and efficiency adjusted PCR values (shown as 40-Ct) for fractions of samples GC-26/66 and GC-55/68, respectively, subjected to fractionation to look at membrane protection of SARS-CoV-2 RNAs. The values for the in-house PCRs detecting 7a subgenomic RNA only and 7a total RNA (i.e. genomic RNA and subgenomic RNA up to and including 7a) are shown as blue and red bars, respectively. The different fractions are indicated on the X-axis.

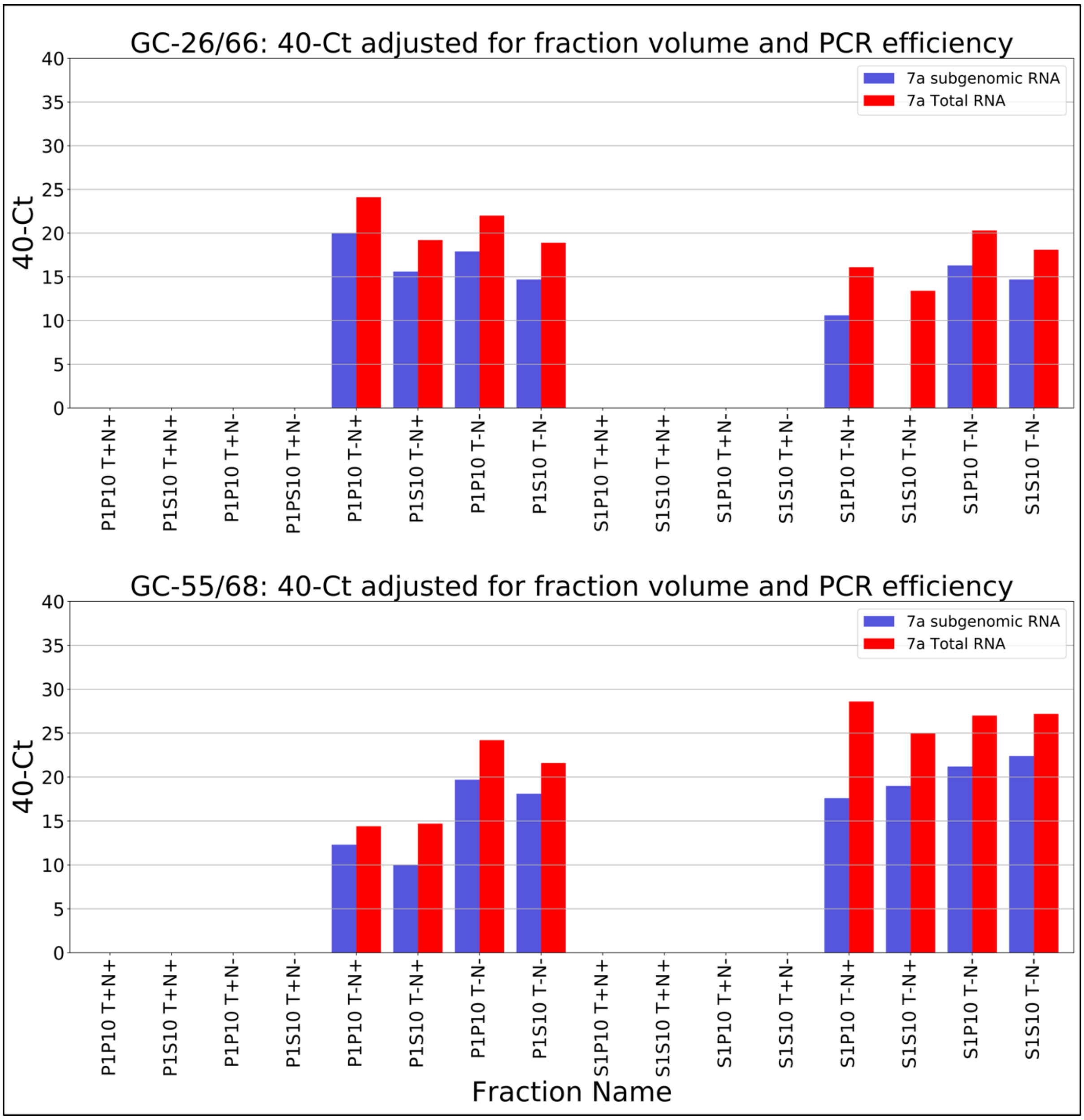

Supplement: Supplementary file 1 — Supplementary Information [file 41467_2020_19883_MOESM1_ESM.pdf]
