# Supplementary material for: SARS-CoV-2 genomic and subgenomic RNAs in diagnostic samples are not an indicator of active replication
Source: Nat Commun. 2020 Nov 27;11:6059. doi: 10.1038/s41467-020-19883-7 (PMC7695715; doi:10.1038/s41467-020-19883-7)
Supplement: Supplementary file 3 — Description of Additional Supplementary Information [file 41467_2020_19883_MOESM3_ESM.pdf]

**Description of Additional Supplementary Files**

**File Name**: Supplementary Data 1

**Description:** Fasta file (Wuhan-Hu-1-NC_045512-21500-and-subgenomics-SA4.fasta) with the sequences used to map genome and subgenomic RNAs.
